# Limb-sparing reconstruction of acral lentiginous melanoma on the plantar foot using biodegradable temporizing matrix and split-thickness skin grafting: a case report

**DOI:** 10.1093/jscr/rjaf956

**Published:** 2025-11-25

**Authors:** Louis Boyce, Krzysztof Sosnowski, Yash Verma, Maharukh Daruwalla

**Affiliations:** Lister Hospital, East and North Hertfordshire NHS Trust, Coreys Mill Lane, Stevenage SG1 4AB, United Kingdom; School of Clinical Medicine, University of Cambridge, Hills Rd, Cambridge CB20SP, United Kingdom; Lister Hospital, East and North Hertfordshire NHS Trust, Coreys Mill Lane, Stevenage SG1 4AB, United Kingdom; Lister Hospital, East and North Hertfordshire NHS Trust, Coreys Mill Lane, Stevenage SG1 4AB, United Kingdom

**Keywords:** limb-sparing reconstruction, dermal substitutes, acral lentiginous melanoma, biodegradable temporizing matrix

## Abstract

Acral lentiginous melanoma, a rare and aggressive melanoma subtype, often affects weight-bearing areas like the sole of foot, presenting complex reconstructive challenges after wide local excision. An 87-year-old female chiropodist with a 4.5-cm, 1-mm thick ALM on her left ball of foot underwent wide local excision with 1-cm margins, preserving hallux neurovascular bundles and achieving complete excision. Reconstruction utilized a biodegradable temporizing matrix (BTM) with negative pressure wound therapy for 5 weeks, followed by split-thickness skin grafting (SSG) after BTM delamination. She remained non-weight-bearing during recovery and regained full foot function long-term. BTM with SSG facilitates robust neodermis formation, offering an effective alternative to flap reconstruction, especially in elderly patients or defects with exposed bone or tendon, avoiding donor site morbidity while ensuring favorable functional outcomes with early ambulation and low complication rates. This approach highlights a shift toward limb-sparing techniques over amputation for plantar ALM in appropriately selected patients.

## Introduction

Acral lentiginous melanoma (ALM) is a rare subtype of malignant melanoma, comprising 2%-3% of all melanoma diagnoses [1]. It predominantly affects the palms, nailbeds, and soles of the feet: [2] areas subject to unique biomechanical stresses. The atypical presentation and anatomical location of ALM often delay its recognition, resulting in diagnosis at advanced stages. Wide local excision (WLE) remains the gold standard for treatment, with oncologic margins of 1–2 cm determined by Breslow thickness to ensure complete tumor clearance [2]. However, the resultant defect, particularly when excising a lesion in a weight-bearing area like the plantar surface of the foot, poses a complex reconstructive challenge.

In the past, advanced ALM cases frequently necessitated major amputations, such as below-knee or ray amputations, to address the difficulties of reconstructing large defects in high-pressure zones. Although amputations ensure oncologic control, they often impair function, diminish quality of life, and increase morbidity, particularly in older patients. Modern reconstructive surgery has shifted toward limb preservation, employing techniques like dermal substitutes, local flaps, and free tissue transfer. Dermal matrices, such as biodegradable temporizing matrix (BTM) and Integra®, have proven effective by promoting neodermis formation and supporting subsequent skin grafting [3]. These methods strive to reconcile oncologic clearance with functional recovery, especially for patients with comorbidities or those unfit for intricate microsurgical interventions.

This case report details the successful treatment of a plantar ALM in an elderly patient using WLE, followed by BTM and delayed split-thickness skin grafting (SSG). The approach demonstrates the value of dermal substitutes in limb-sparing reconstruction and highlights the critical role of multidisciplinary collaboration in optimizing patient outcomes.

## Case report

An 87-year-old female chiropodist presented to clinic with an irregularly pigmented lesion, rapidly progressing over 1 month, on the ball of her left foot, near the first metatarsophalangeal joint. The lesion measured 4.5 cm in diameter and displayed variegated pigmentation, poorly defined borders, and skip areas. She reported discomfort during weight-bearing but no bleeding or systemic symptoms. Her medical history included asthma, psoriasis, hypothyroidism, obesity, and previous malignant lesions on both feet. As a chiropodist, she was highly active, and the importance of preserving foot function for her professional and personal mobility was vital.

A 4 mm punch biopsy confirmed ALM with a Breslow thickness of 1 mm. Physical examination revealed no palpable lymphadenopathy in the inguinal or popliteal regions, and staging computed tomography (CT) confirmed no metastases. Following review by the skin multidisciplinary team, WLE with 1 cm margins was recommended.

The WLE was performed under spinal anesthesia with a left ankle block. Intravenous co-amoxiclav was administered at induction, and the foot was double-prepped with betadine and alcoholic chlorhexidine. The lesion was excised superficial to the neurovascular bundles of the left great toe, resulting in a 5.5 × 4.5 cm defect exposing the plantar fascia. Histopathology confirmed complete excision with no residual tumor. BTM was applied intraoperatively, inset with 3–0 nylon sutures, quilted with staples, and covered with a Mepitel dressing and negative pressure wound therapy (vaccum assisted closure/ VAC) at 125 mmHg continuous suction. A plaster of Paris (POP) backslab was applied to immobilize the foot.

**Figure 1:**
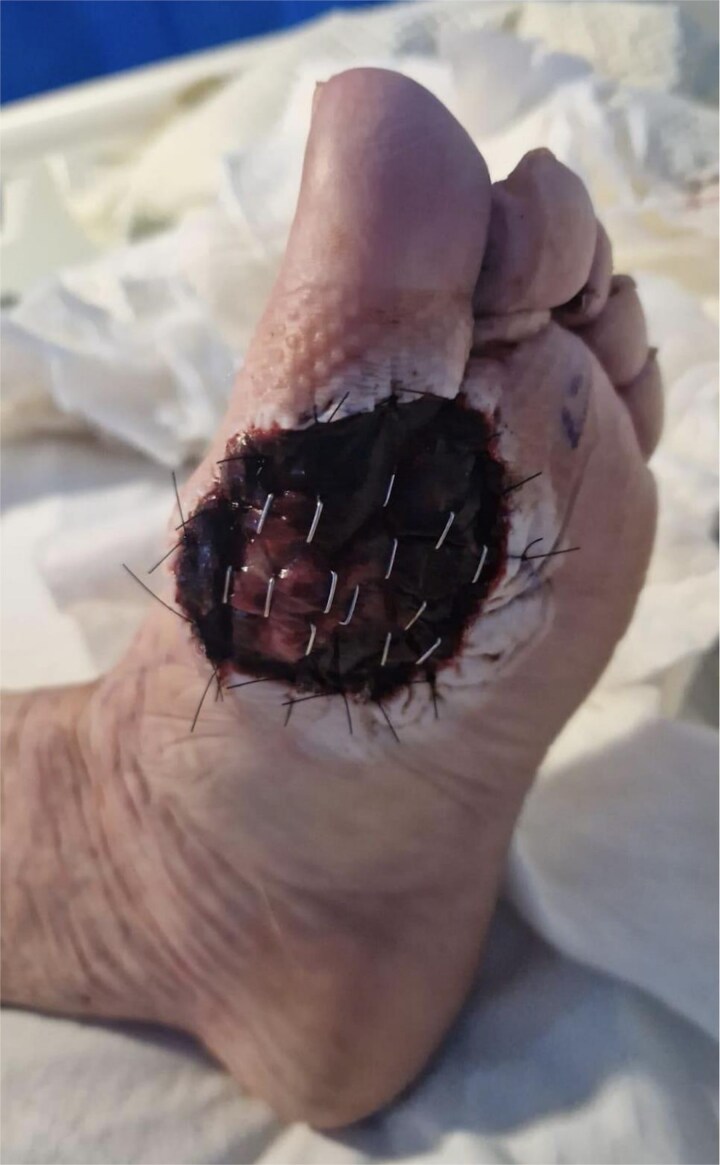
WLE of left ball of foot ALM with BTM *in situ* 1 week post-operatively.

The patient was admitted overnight for physiotherapy assessment, as non-weight-bearing status was required for 5 weeks. She continued intravenous antibiotics as an inpatient, transitioning to oral antibiotics upon discharge the following day. Venous thromboembolism (VTE) prophylaxis was maintained for 5 weeks, with weekly outpatient dressing changes. At 1-week follow-up, the backslab was removed, and the VAC was inspected, confirming BTM adherence (Fig. 1).

**Figure 2:**
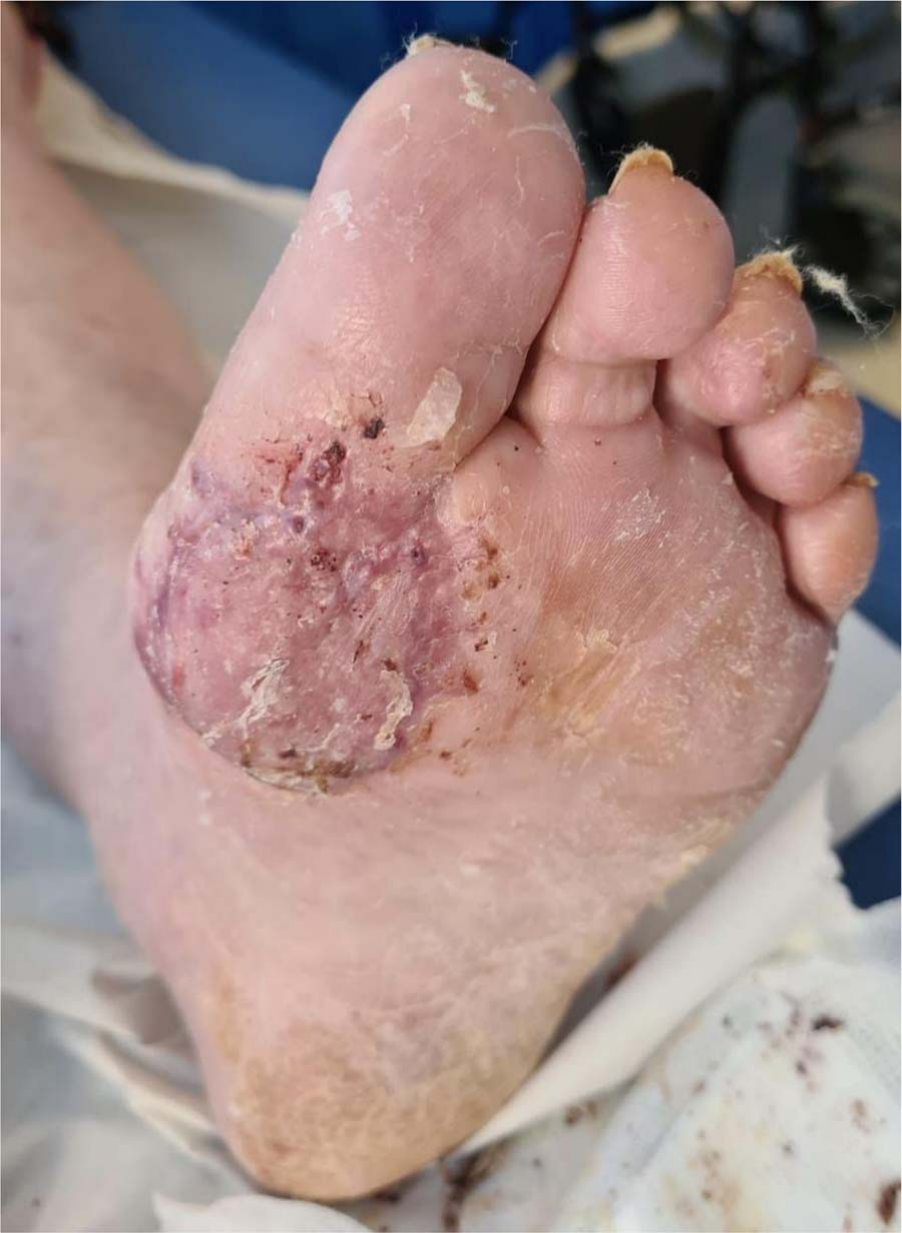
BTM delaminated and SSG inset overtop 1 week post-operatively.

**Figure 3:**
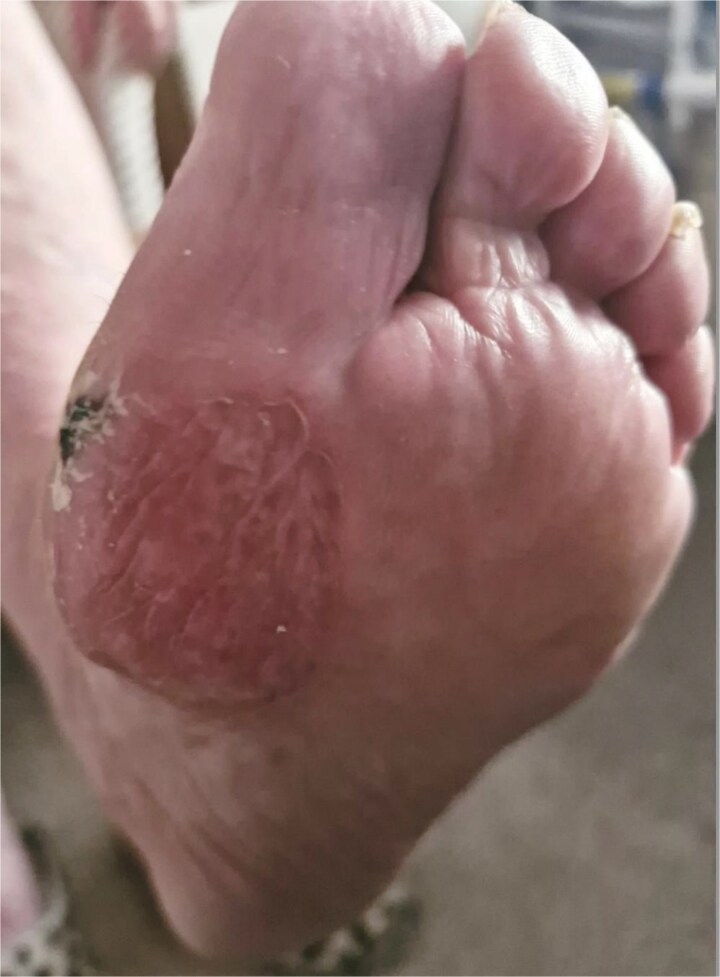
Healed SSG over the left ball of the foot 10 months post-reconstruction.

Five weeks post-WLE, the BTM showed robust integration with a vascularized neodermis. Under spinal anesthesia, with intravenous antibiotics at induction, the silicone layer was delaminated, and a 0.3 mm split-thickness skin graft (harvested from the left thigh) was applied (Fig. 2). The graft was secured with a Jelonet tie-over dressing and a POP backslab. VTE prophylaxis continued for 10 days, and the patient continued her rehabilitation. No immediate complications were observed. At 2 months post-grafting, the patient achieved full weight-bearing capacity and resumed her professional activities (Fig. 3).

## Discussion

This case illustrates a successful limb-sparing and function-preserving approach to managing a plantar ALM. The novel aspect of this report is the reconstruction of a weight-bearing, glabrous plantar skin defect using NovoSorb BTM after melanoma excision, preserving function in an elderly patient.

The plantar surface of the foot presents unique reconstructive challenges due to its role in weight-bearing and limited local tissue availability. Traditional reconstructive options, such as local or free flaps, often involve significant donor-site morbidity or require microsurgical expertise, which may not be suitable for elderly patients or those with comorbidities. Major amputation, while historically common, is associated with prolonged rehabilitation, prosthetic challenges, and reduced quality of life.

Dermal substitutes like BTM and Integra® have revolutionized reconstruction in such scenarios. BTM provides a temporary scaffold that supports neodermis formation while minimizing contracture and infection risk [3]. A 20-year literature review highlights the growing adoption of dermal matrices for complex defects, particularly in acral regions [4]. For plantar defects, BTM facilitates the formation of a durable, flexible neodermis capable of withstanding mechanical stress, as demonstrated in our patient’s ability to resume professional activities and in other similar case series [5–7].

Compared to flap-based reconstruction, BTM avoids donor-site morbidity, reduces operative time, and eliminates the need for microsurgical expertise. In elderly patients, where comorbidities may preclude complex procedures, BTM provides a less invasive yet effective alternative. However, challenges include the need for staged procedures and prolonged wound care, which require patient compliance and multidisciplinary oversight.

Limitations of BTM include cost and availability, which may restrict its use in resource-limited settings [8]. Additionally, while suitable for defects with exposed fascia, BTM may be less effective for defects involving bone or tendon, where flap reconstruction remains the gold standard. Future research should focus on optimizing BTM protocols, including the timing of grafting.

## Conclusion

This case demonstrates the efficacy of BTM with delayed SSG as a safe, effective, and function-preserving technique for reconstructing plantar ALM defects. By achieving oncologic clearance and restoring full foot function, this approach exemplifies the shift toward limb salvage in carefully selected patients. As reconstructive techniques evolve, dermal substitutes will likely play an increasingly prominent role in managing complex acral defects, offering a viable alternative to amputation or flap-based reconstruction.
